# 4T1-derived exosomes downregulate NKG2D expression on NKp46^+^ splenocytes

**DOI:** 10.1016/j.bbrep.2026.102874

**Published:** 2026-11-06

**Authors:** Rizan Fallahzadeh, Motahare Khasteband, Jafar Rezaie, Adel Mohammadzadeh

**Affiliations:** a Department of Microbiology and Virology, School of Medicine, Urmia University of Medical Sciences, Urmia, Iran; b Solid Tumor Research Center, Cellular and Molecular Medicine Research Institute, Urmia University of Medical Sciences, Urmia, Iran; c Cellular and Molecular Research Center, Cellular and Molecular Medicine Research Institute, Urmia University of Medical Sciences, Urmia, Iran; d Department of Immunology and Genetics, School of Medicine, Urmia University of Medical Sciences, Urmia, Iran

**Keywords:** Texosome, NKG2D, Flow cytometry, qRT-PCR, FasL, IFN-γ

## Abstract

Tumor-derived exosomes can reshape anti-tumor immunity within the tumor microenvironment (TME), but their effects on the expression of NKG2D remain poorly defined. Here, we examined how exosomes from the 4T1 murine breast cancer cell line (Texosomes) affect NKp46^+^ splenocytes.

Murine splenocytes were co-cultured with Texosomes, and NKG2D expression was measured by flow cytometry. Transcript levels of NKG2D, IFN-γ, and FasL were quantified by qRT-PCR, and splenocyte proliferation was evaluated by CFSE assay.

Our results showed that Texosomes significantly reduced NKG2D expression on NKp46^+^ cells (P < 0.01), accompanied by decreased transcription of NKG2D, IFN-γ, and FasL (P < 0.01) and a marked reduction in splenocyte proliferation. These findings showed that NKG2D expression on NKp46^+^ splenocytes was reduced in splenocytes exposed to Texosomes, supporting further investigation of the involvement of Texosomes on immune modulation in tumor settings.

## Highlights

• 4T1-derived exosomes, Texosomes, reduce NKG2D expression on NKp46^+^ splenocytes.
• Texosomes reduce NKG2D, IFN-γ, and FasL transcript levels in splenocytes.
• Texosomes suppress splenocyte proliferation in vitro.

## 1 Introduction

Immune cells are fundamental to orchestrating effective anti-tumor responses [1]; however, their functionality is frequently compromised within the tumor microenvironment (TME), leading to attenuated immune surveillance [2]. The recruitment of anti-inflammatory cells, such as regulatory T cells (Tregs) and myeloid-derived suppressor cells (MDSCs), has led to the TME being characterized as an immunocompromised field [3]. Tumor cells exploit extracellular vesicles (EVs)—specifically tumor-derived exosomes, hereafter referred to as Texosomes [4]. Exosomes are phospholipid-bilayer structures, typically 30–150 nm in diameter, which encapsulate a diverse cargo of proteins, lipids, and microRNAs (miRNAs). Beyond their roles in intercellular communication, signaling, and metabolic regulation, these vesicles are increasingly recognized as reliable prognostic biomarkers [5], [6], [7]. Standardized markers for exosome characterization include CD63, CD81, CD9, TSG-101, and ALIX [4]. Tumor-derived exosomes disrupt anti-tumor immunity by impairing innate immune effectors, in part through the delivery of immunosuppressive cargo such as TGF-β, PD-L1, and TRAIL [8].

Natural killer (NK) cells are indispensable for tumor immunosurveillance [9]. Unlike CD8^+^ T cells, which recognize antigens presented on MHC molecules, NK cells employ a "missing-self" strategy to identify and eliminate transformed or virus-infected cells that have downregulated MHC class I expression. This strategy is governed by a balance of germline-encoded activating and inhibitory receptors, including the killer-cell immunoglobulin-like receptors (KIRs) [10]. Among the activating receptors, NKG2D serves as a sentinel for stress-induced ligands such as MICA and MICB, which are frequently upregulated on malignant cells. NKG2D is broadly expressed across cytotoxic lineages, including NK cells, CD8^+^ T cells, and γδ T cells, highlighting its central role in tumor immunity [11]. Notably, tumor-derived exosomes can display NKG2D ligands such as MICA/B and ULBP1/2 on their surface, and this exosomal ligand presentation has been shown to downregulate NKG2D receptor expression and impair NK cell-mediated cytotoxicity in vitro [12]. Despite the well-established association between TME-driven immune exhaustion and disease progression [13], the specific impact of Texosomes on NKG2D expression, downstream effector gene transcription, and the proliferative capacity of NKp46^+^ splenocytes remains incompletely defined.

In this study, we investigated the modulatory effects of 4T1-derived Texosomes on murine splenocytes, focusing on three complementary aspects of NK cell-mediated immunity. First, we assessed NKG2D expression on NKp46^+^ splenocytes following Texosome exposure. Second, we evaluated the transcriptional expression of key effector genes, including IFN-γ and FasL; IFN-γ is a central mediator of broad immune activation, and FasL is a core component of the cytotoxic machinery used by NK and CD8^+^ T cells to induce tumor cell apoptosis [14], [15], [16]. Finally, we examined the anti-proliferative effects of Texosomes on splenocytes in order to build a comprehensive picture of how Texosomes reshape the phenotype and proliferative output of this critical anti-tumor effector population.

## 2 Materials and methods

### 2.1 Cell culture

The murine breast cancer cell line 4T1 (ATCC number: CRL-2539) was purchased from the Pasteur Institute of Iran and tested for mycoplasma contamination. Cells were cultured in T75 tissue culture flasks containing RPMI-1640 medium supplemented with 10% heat-inactivated fetal bovine serum (FBS) (Gibco, UK), 1% penicillin-streptomycin (Gibco, UK), and 2 mM l-glutamine (Sigma, USA), and incubated at 37 °C with 5% CO_2_.

### 2.2 Isolation and characterization of Texosomes

The 4T1 cell line was cultured in RPMI-1640 medium supplemented with 10% FBS. To minimize bovine exosome contamination, when cells reached approximately 80% confluency, the culture medium was replaced with serum-free RPMI-1640 for 48 h. The conditioned medium was then harvested for Texosome isolation using the Exocib kit (Exocib, Iran) according to the manufacturer's instructions.

Briefly, 10 mL of the harvested supernatant was centrifuged at 300×g for 20 min at 4 °C to pellet cellular debris. The resulting supernatant was passed through a 0.22 μm syringe filter (SPL, Korea) to remove larger vesicles and remaining contaminants. Texosomes were concentrated by adding the provided PEG-based precipitation reagent and incubating overnight at 4 °C with constant agitation. Following incubation, the samples were centrifuged at 300×g for 60 min. The resulting exosomal pellet was resuspended in 100 μL of the kit's designated washing buffer (Reagent B).

To ensure experimental reproducibility, the total protein concentration of the isolated Texosomes was determined using a BCA protein assay kit (KPG-Iran). Texosome samples were normalized to equivalent protein concentrations prior to all downstream assays and stored at −80 °C until required.

### 2.3 Western blot analysis of Texosomes

To characterize Texosomes, protein from Texosomes was extracted by lysis in RIPA buffer and equal amounts of protein (20 μg per lane), quantified by BCA assay as described above, were mixed with Laemmli sample buffer, denatured at 95 °C for 5 min, and separated by 12% SDS-PAGE. Proteins were then transferred onto PVDF membranes. Membranes were blocked with 5% BSA in TBS-Tween (TBST) for 1h at room temperature, then incubated overnight at 4 °C with the following primary antibodies (Santa Cruz Biotechnology, Santa Cruz, CA, USA) diluted in TBST: anti-CD9 (C-4; sc-13118, 1:300), anti-CD63 (MX-49.129.5; sc-5275, 1:500), anti-CD81 (B-11; sc-166029, 1:400), and anti-β-actin (sc-47778, 1:300). After washing three times for 10 min each with TBST, the membranes were incubated for 1h at room temperature with the corresponding HRP-conjugated secondary m-IgGκBP-HRP (sc-516102; 1:2000) and anti-rabbit IgG-HRP (Sc-2357; 1:1000 in TBST). Following 2 times washing, bands were visualized using an enhanced chemiluminescence (ECL) kit and imaged with a camera and exposed to X-ray film.

### 2.4 Flow cytometry analysis of Texosomes

To characterize the isolated Texosomes, the presence of the exosomal surface marker CD63 was evaluated by flow cytometry. Texosomes were incubated with a fluorophore-conjugated anti-CD63 antibody (NVG-2, BioLegend, USA) or an appropriate isotype control for 30 min at 4 °C in the dark. Following incubation, the samples were diluted in an appropriate physiological buffer (PBS containing 0.1% BSA) and ultracentrifuged at 100,000×g for 30 min at 4 °C to remove unbound antibodies and concentrate the labeled vesicles. The stained Texosomes were resuspended in 200 μL of filtered buffer and analyzed using a BD FACSCalibur. Flow cytometry data were processed and analyzed using FlowJo software (version X, USA). Fluorescence intensity was compared against an unstained control to establish baseline background levels and ensure the specificity of the CD63 signal.

### 2.5 Cell isolation and culture with Texosomes

Spleens from BALB/c mice (n = 3; see Ethics Statement) were dissociated by mechanical disruption using a syringe plunger. The resulting cell suspension was centrifuged at 300×g for 5 min, resuspended in PBS, treated with RBC lysis buffer to eliminate red blood cells, and then washed twice with PBS. Next, the cells were counted and cultured in 6-well plates in complete RPMI-1640 medium supplemented with 10% FBS and 1% penicillin-streptomycin (Gibco, UK). After 24 h, 50 μg/mL of Texosomes was added to the plates, and the cells were used for flow cytometry and mRNA isolation 24 h and 48 h later, respectively.

### 2.6 Flow cytometry analysis for immunophenotyping of splenocytes

In this study, we used splenocytes from BALB/c mice (6–8 weeks old). The harvested cells were plated with or without Texosomes in the presence of the immune cell stimulator concanavalin A (ConA; 5 μg/mL, Sigma).

For NKG2D expression on NKp46+ cells, bulk splenocytes were seeded at 1 × 10^6^ splenocytes per well in 6-well plates and cultured in the presence or absence of 4T1-derived Texosomes at 50 μg/mL for 24 h at 37 °C. Subsequently, the cells were stained with anti-NKp46-PerCP-Cy5.5 and anti-NKG2D-PE antibodies (both from BioLegend, USA) to distinguish NKp46^+^ splenocytes from other immune cells. The collected data were analyzed using FlowJo software (FlowJo, USA).

### 2.7 CFSE staining

To examine the effect of Texosomes on proliferation capacity of splenocytes, we used a CFSE kit (BioLegend, USA). Bulk splenocytes were seeded for splenocyte proliferation (CFSE dilution) at 1 × 10^5^ splenocytes per well in 6-well plates and cultured in the presence or absence of 4T1-derived Texosomes at 50 μg/mL for 72 h. According to the manufacturer's protocol, a 5 μM CFSE working solution was prepared. The splenocytes were incubated with it at 37 °C for 20 min, the reaction was quenched with complete RPMI-1640 medium, and the cells were cultured with 50 μg/mL of Texosomes for 3 days. Cells were then collected, acquired on a FACSCanto (Becton Dickinson, USA), and analyzed using FlowJo software (Treestar Inc., USA).

### 2.8 RNA isolation and qPCR analysis

After culturing the bulk spleen-derived immune cells (1 × 10^6^ cells/well) with 50 μg/mL of Texosomes for 48 h, total RNA was isolated and purified using an RNA isolation kit (FavorGen, Taiwan) according to the manufacturer's protocol. RNA concentrations were measured using a NanoDrop (Thermo Fisher Scientific). RNA was then reverse-transcribed into complementary DNA (cDNA) using a cDNA synthesis kit (GeneAll, Korea). The cDNA was quantified using 2× SYBR Green RT-PCR Mix (Ampliqon, Sweden) in a thermal cycler (MIC qPCR cycler, Bio Molecular Systems). Fold changes in gene expression were calculated using the 2^−ΔΔCt method [17]. The following primers were used: for NKG2D, GGCAGTCACCTTTCTTCTCTT (forward) and GGCTACTGATAACCACATCTACTG (reverse); for IFN-γ, GTGAACAACCCACAGATCCAG (forward) and CCAGAATCAGCACCGACT (reverse); for FasL, GCCATCACAACCACTCCC (forward) and TCCTAATCCCATTCCAACCAG (reverse); and for GAPDH, AACGACCCCTTCATTGACCT (forward) and ATGTTAGTGGGGTCTCGCTC (reverse).

### 2.9 Statistical analysis

Data were analyzed using GraphPad Prism (GraphPad Software). Data are presented as mean ± SEM of three independent biological replicates (n = 3 mice per group), with each value representing the mean of technical triplicate measurements for that mouse. The number of independent biological replicates (n = 3) was used for all statistical comparisons; technical replicates were averaged and were not treated as independent observations. Comparisons among multiple groups were performed by one-way analysis of variance (ANOVA) followed by Tukey's multiple-comparison test and statistical significance is indicated as *P < 0.05, **P < 0.01, ***P < 0.001, and ****P < 0.0001; 'ns' indicates not significant. And differences were considered statistically significant at P < 0.05.

## 3 Results

### 3.1 Characterization of Texosomes

The physical and molecular properties of the isolated Texosomes were validated using morphological analysis and protein expression profiling. Morphological assessment by scanning electron microscopy (SEM) (Fig. 1A) and transmission electron microscopy (TEM) (Fig. 1B) revealed that the isolated vesicles exhibited the characteristic cup-shaped morphology and spherical structure typical of exosomes, with diameters (determined by ImageJ software) within the expected range (30–150 nm). Furthermore, the identity of the isolated vesicles was confirmed by western blot analysis (Fig. 1C) and flow cytometry for CD63 (Fig. 1D), which demonstrated the robust presence of the classical exosomal protein markers CD63, CD81, and CD9. These results collectively confirm an exosome-enriched vesicle preparation from the 4T1 cell-conditioned medium.

**Fig. 1:**
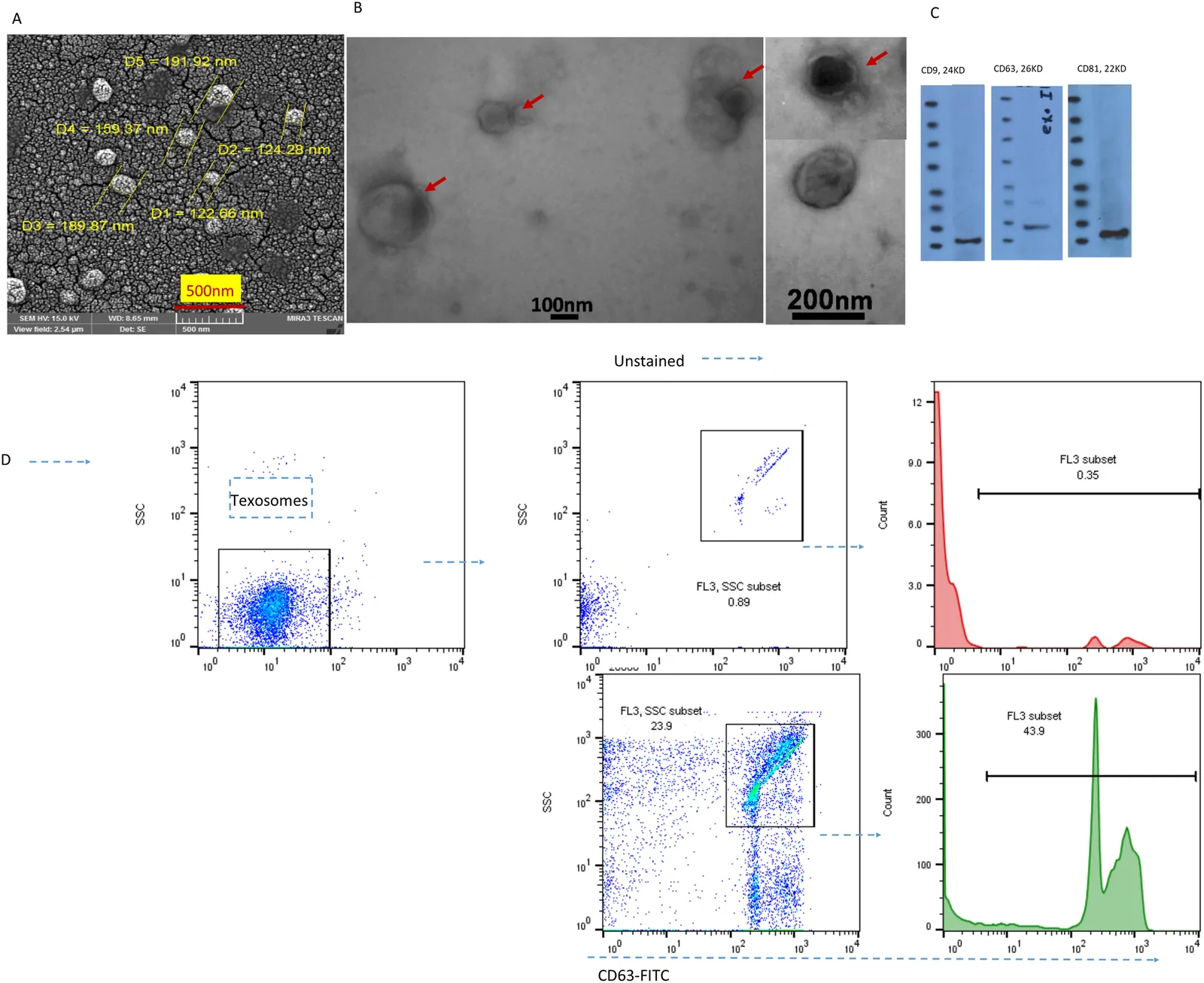
Characterization of Texosomes. The mouse 4T1 breast cancer cell line was cultured in serum-free RPMI-1640 for 48 h, and Texosomes were isolated from the supernatant and analyzed by scanning electron microscopy (SEM) (A) and transmission electron microscopy (TEM) (B). Western blotting was used to detect CD9, CD81, and CD63 expression (C). The percentage of CD63-positive Texosomes was quantified by flow cytometry, showing higher expression than in the unstained control (D).

### 3.2 Inhibition of NKG2D expression on NKp46+ eplenocytes by Texosomes: Evidence from co-culture experiments

NKG2D is recognized as a powerful activating receptor on NK cells and CD8^+^ T cells [18]. In light of this, we investigated how Texosomes might influence NKG2D expression on NKp46^+^ splenocytes. In bulk splenocyte cultures, NKG2D expression was lower in Texosome-treated cells than in control cultures.

As illustrated in Fig. 2A, we compared concanavalin A-activated splenocytes with and without Texosomes. After 24 h of co-culture, NKG2D expression was significantly diminished (P < 0.01) in the Texosome-treated group compared with the untreated group (Fig. 2A).

**Fig. 2:**
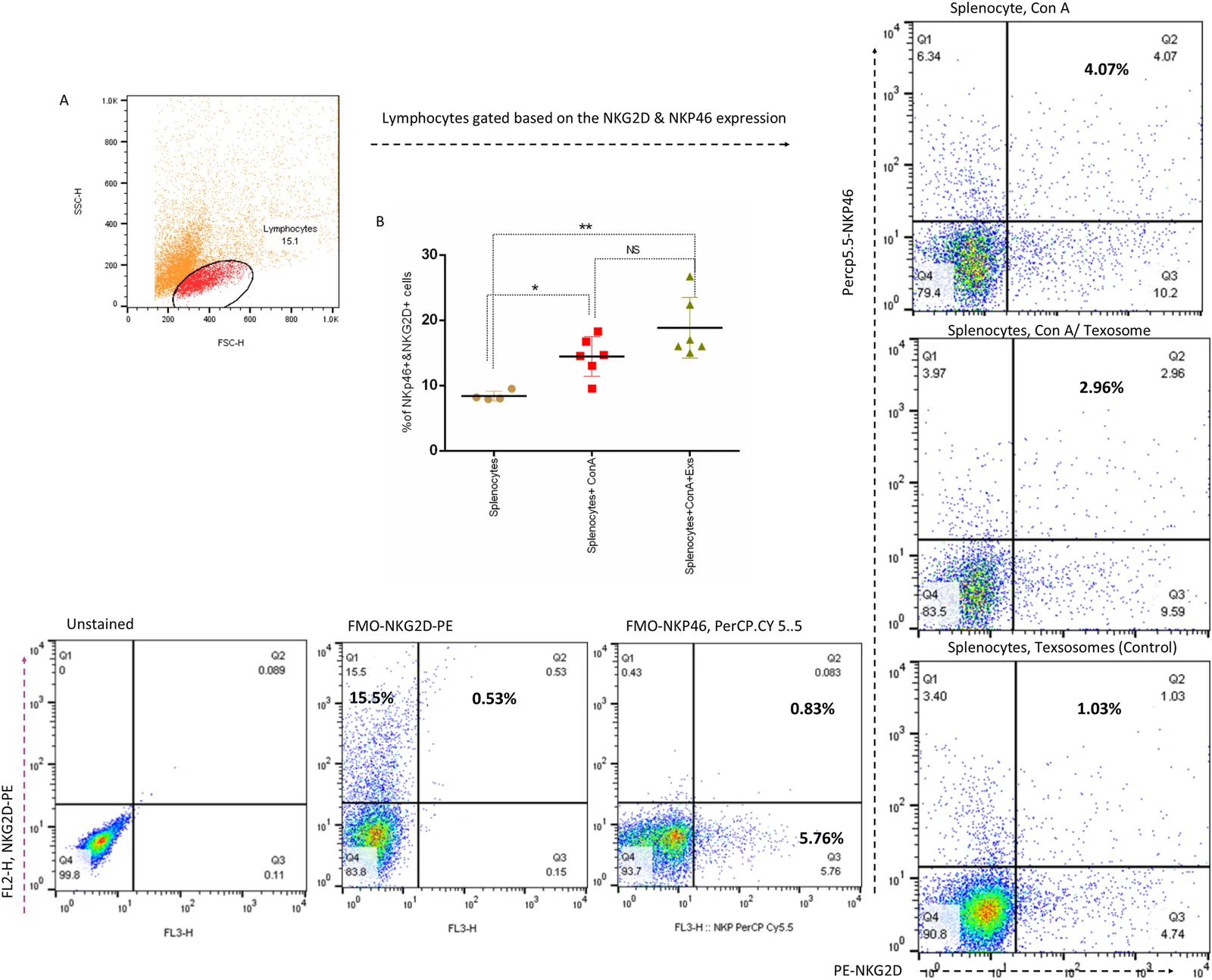
Texosomes inhibit NKG2D expression on NKp46^+^ splenocytes. Splenocytes from BALB/c mice (1 × 10^6^ cells) were co-cultured with Texosomes 50 μg/mL for 24 h at 37 °C and then stained with anti-NKp46-PerCP-Cy5.5 and anti-NKG2D-PE antibodies. Representative flow cytometry histograms (A) and the percentage of NKG2D^+^ cells within the NKp46^+^ population (B) show a significant decrease in NKG2D expression on NKp46^+^ splenocytes. Three BALB/c mice were used, and the cells from each mouse were analyzed in triplicate. Fluorescence intensity was compared against unstained and fluorescence-minus-one (FMO) controls to establish background levels and confirm the specificity of the NKG2D and NKp46 signals; the FMO and negative controls are shown in the bottom-left panel. Results are expressed as mean ± SEM and were analyzed by one-way ANOVA with Tukey's multiple-comparison test. Statistical significance is indicated as *P < 0.05, **P < 0.01, ***P < 0.001, and ****P < 0.0001; 'ns' indicates not significant.

### 3.3 4T1-derived Texosomes lower the splenocyte proliferation in vitro

To determine whether Texosomes modulate immune cell proliferation, CFSE-labeled splenocytes were stimulated with ConA in the presence or absence of 50 μg/mL Texosomes for 72 h. Proliferation was assessed by flow cytometric analysis of CFSE dye dilution. As expected, unstimulated splenocytes retained a single, high-intensity CFSE peak, confirming the absence of cell division at baseline (splenocyte control; CFSE-high), whereas ConA-stimulated splenocytes exhibited robust proliferation, evidenced by progressive CFSE dilution across multiple daughter generations. Splenocyte proliferation was lower in cultures treated with 4T1-derived Texosomes 50 μg/mL than in untreated control cultures (Fig. 3; P < 0.001). Together, these observations are consistent with an association between 4T1-derived Texosomes and reduced NKG2D expression; however, as these experiments used bulk splenocyte cultures without functional NK-cell assays, they do not establish a direct inhibitory mechanism.

**Fig. 3:**
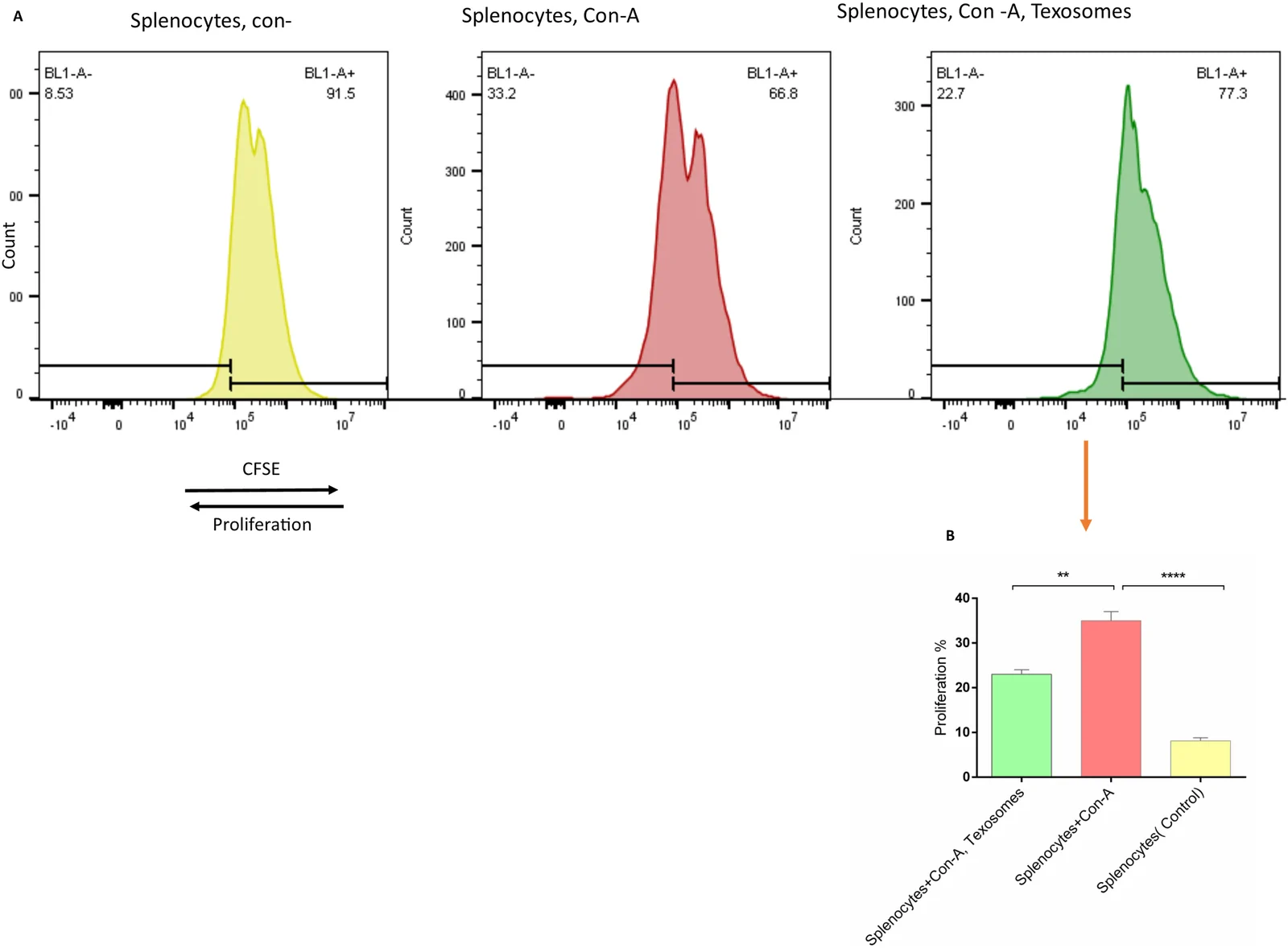
4T1-derived Texosomes exert inhibitory effects on immune cell proliferation. To explore the effect of Texosomes on immune cells, splenocytes were incubated with 50 μg/mL of Texosomes, and after 3 days, cell proliferation in the different groups was assessed by flow cytometry using CFSE dye (A, B). Representative histograms show a leftward shift of CFSE fluorescence intensity in the proliferating populations for each condition. Three BALB/c mice were used, and the cells from each mouse were analyzed in triplicate. Error bars indicate mean ± SEM. P-values were calculated using one-way ANOVA with Tukey's multiple-comparison test. Statistical significance is indicated as *P < 0.05, **P < 0.01, ***P < 0.001, and ****P < 0.0001; 'ns' indicates not significant.

### 3.4 The regulatory effects of Texosomes on splenocytes: Insights into downregulation of NKG2D, FASL, and IFN-γ

To explore the effects of Texosomes on gene expression in splenocytes, we measured the expression of NKG2D, FasL, and IFN-γ in immune cells exposed to Texosomes. Our experiments showed that Texosomes modulate the expression of IFN-γ, NKG2D, and FasL. Compared with untreated control cultures, NKG2D expression was lower in Texosome-treated splenocytes (P < 0.001), as were FasL (P < 0.05) and IFN-γ (P < 0.01) (Fig. 4). GAPDH was used as an internal control.

**Fig. 4:**
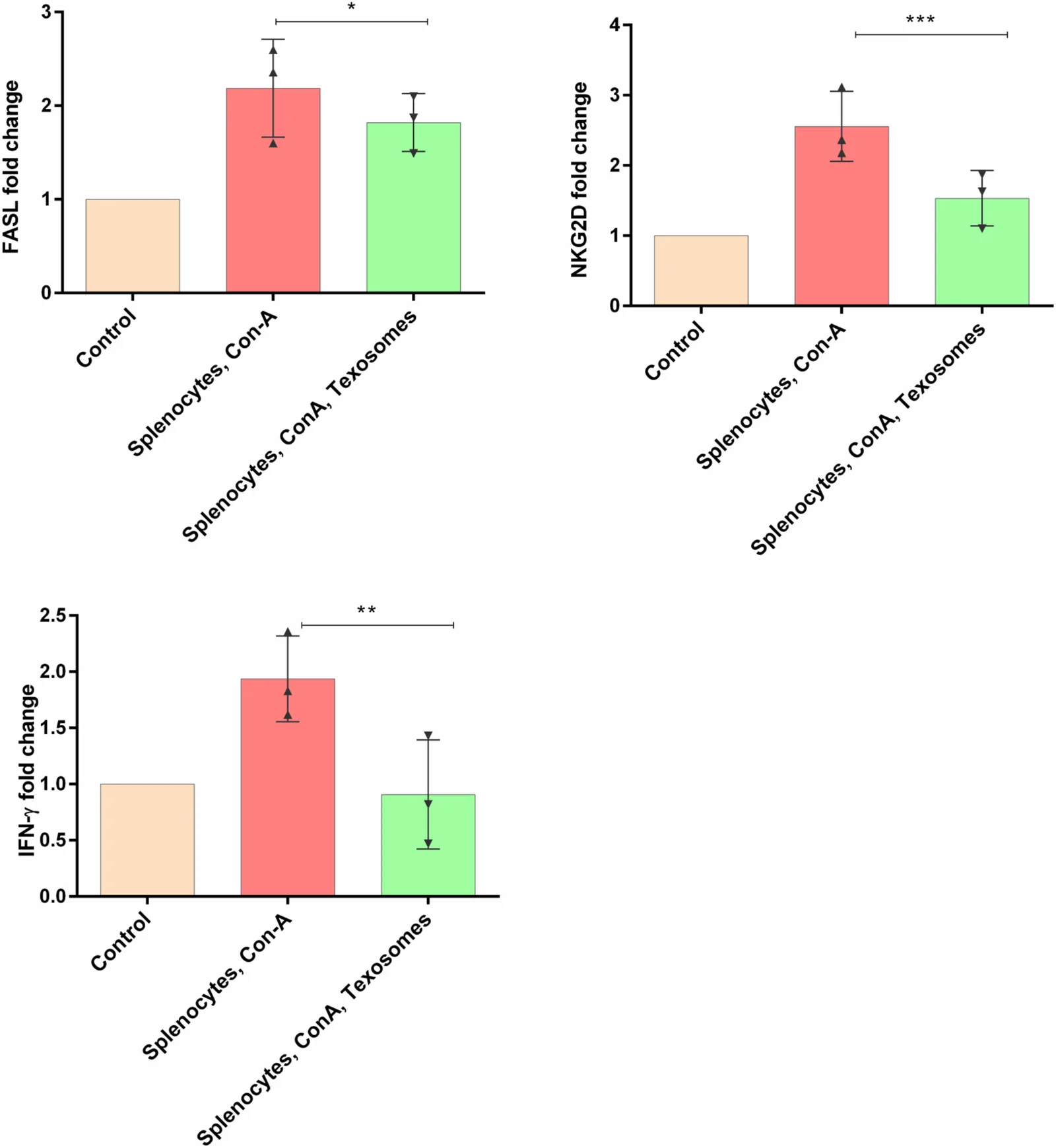
Texosomes modulate gene expression in splenocytes. To assess gene expression patterns in immune cells, total RNA was extracted from splenocytes and used for cDNA synthesis according to the kit instructions. qRT-PCR was used to measure the expression levels of NKG2D, IFN-γ, and FasL. Results are presented as fold change relative to the housekeeping gene GAPDH. Error bars indicate mean ± SEM. P-values were calculated using one-way ANOVA with Tukey's multiple-comparison test. Statistical significance is indicated as *P < 0.05, **P < 0.01, ***P < 0.001, and ****P < 0.0001; 'ns' indicates not significant.

## 4 Discussion

In this study, we showed that 4T1-derived Texosomes significantly reduce NKG2D expression on NKp46^+^ splenocytes and modulate the mRNA expression of key cytotoxic effector molecules, specifically NKG2D, FasL, and IFN-γ, in splenocytes. Using a CFSE dilution assay, we showed that Texosome-treated splenocytes had reduced proliferative capacity compared with stimulated controls, suggesting that Texosomes not only reduced NKG2D expression on NKp46^+^ population of splenocytes but also reduced proliferation in the bulk splenocyte population. Together, these findings showed that Texosomes were associated with observed regulatory effects in our in vitro splenocyte model, disrupting both the transcriptional program and the proliferative output of effector immune cell populations. Additionally, our findings at the mRNA level showed that the modulation of FasL and IFN-γ, both downstream products of NK and T cell responses against tumor antigens, also supports the observed regulatory effects of Texosomes.

NKG2D functions as a critical stress-sensing receptor on NK cells, CD8^+^ T cells, and γδ T cells, recognizing ligands such as MICA/B and ULBPs that are upregulated on transformed or stressed cells [19], [20]. Engagement of this receptor is required not only for direct cytotoxic killing but also for downstream proliferative and effector responses.

Exosomes are integral mediators of intercellular communication, regulating diverse physiological and pathological processes, including cell proliferation, differentiation, and survival [21]. In this study, we investigated the effect of Texosomes on NKG2D expression in NKp46^+^ splenocytes, which are critical population of cytotoxic effectors in anti-tumor immunity. Emerging evidence indicates that the TME actively inhibits the activation, proliferation, and effector functions of cytotoxic lymphocytes [22]. This immunosuppressive setting is maintained through a complex interplay of cellular and biochemical factors.

Although our study did not directly assess metabolic reprogramming, the reduced proliferative capacity of Texosome-treated splenocytes is consistent with a broader pattern in which Texosome cargo, as part of the TME, may affect the anti-tumor immunity of immune cells [23]. For instance, Texosome-encapsulated microRNAs, such as miR-34a, have been implicated in the modulation of the PI3K/AKT signaling axis and are recognized as critical regulators of tumor-immune dynamics. Furthermore, exosomes derived from tumor-associated macrophages (TAMs) and expressing PD-L1 have been shown to modulate the transcription factor PPARα in CD8^+^ T cells. This interaction reprograms lipid metabolism within these effector cells, promoting functional exhaustion, enhancing apoptotic signaling, and ultimately impairing the anti-tumor cytotoxic response in epithelial ovarian cancer (EOC) [24]. A substantial body of literature has established the regulatory role of Texosomes in shaping NK cell responses [25], [26]. It has been shown that Texosome exposure was associated with reduced NKG2D expression on NK cells [27]. It has been also shown that exosomes exert broad immunomodulatory effects by carrying inhibitory molecules such as TGF-β, which increases SMAD2/3 phosphorylation and further dampens NKG2D levels. Notably, Texosomes are known to transport inhibitory molecules such as PD-L1, which suppress immune cells within the TME [24], [28]. Beyond direct modulation, Texosomes can also remodel the TME by inducing the differentiation of Tregs and promoting M2-like polarization of TAMs [29]. For instance, glioma-derived Texosomes enriched with miR-3591-3p have been shown to drive M2-like polarization through modulation of the JAK2/STAT3, PI3K/AKT/mTOR, and MAPK signaling axes [30]. Previous research has demonstrated that tumor-derived exosomes contribute to immune cell exhaustion and promote tumor metastasis [31].

By sensing stress-induced ligands, NK cells provide essential "alert" signals that promote T cell activation and recruitment [32]. However, the TME frequently hijacks this axis to facilitate immune escape. Chronic exposure to tumor-derived inhibitory signals, combined with persistent antigen stimulation, drives progressive exhaustion of cytotoxic lymphocytes, and NKG2D downregulation has emerged as one of the most prominent mechanisms underlying this immune evasion [33]. Notably, suppression of NKG2D on NK cells not only compromises their intrinsic effector function but may also indirectly attenuate T cell responses, thereby exacerbating global immune dysfunction within the tumor site [34], [35].

To investigate the impact of Texosomes on the transcriptional profile of immune cells, we performed quantitative RT-PCR analysis. Our results showed that exposure to Texosomes significantly modulated the expression of key cytotoxic genes, specifically NKG2D, FasL, and IFN-γ. Collectively, these findings showed that, in vitro, Texosome exposure was associated with reduced NKG2D expression on NKp46^+^ cells and with decreased total splenocyte proliferation.

This study has several limitations that should be considered when interpreting our findings. In this study, we used bulk splenocyte populations rather than purified NK or NKp46^+^ subsets, so the observed effects may reflect both direct and indirect (bystander) effects of Texosomes on non-target cell populations. We did not characterize the specific molecular cargo (protein or miRNA) of 4T1-derived Texosomes responsible for the observed modulation in NKG2D expression on the NKp46^+^ splenocytes, which limits our ability to draw direct mechanistic conclusions. Our findings are derived from a single murine tumor model (4T1), and the extent to which these results generalize to other tumor types or to human systems remains to be determined. We measured NKG2D expression on the surface of NKp46^+^ splenocyte subpopulations and gene expression in bulk but did not perform functional assays such as target-cell cytotoxicity, CD107a degranulation, or cytokine production; we therefore cannot conclude that NK-cell effector function was impaired or that the tumor actively escaped NK-cell immunity. In addition, no direct mechanistic link has been established between NKG2D modulation and a specific Texosome cargo component; because our study used only the 4T1 cell line, evaluation in other cell lines and models is also warranted.

Because our analyses for IFN-γ and FasL were limited to mRNA transcript levels, and no corresponding protein measurements were performed, the present results do not establish whether these transcriptional changes are translated into corresponding protein-level or functional changes. Future studies incorporating protein-level and functional assays will be required to confirm these observations. Future high-throughput investigations, such as proteomic and transcriptomic profiling, are essential to fully characterize the molecular cargo of these vesicles. Furthermore, while this study provides insights into the broad immunomodulatory effects of Texosomes on NKp46^+^ splenocytes, future research should use purified immune cell subsets, such as isolated NK and CD8^+^ T cells, to delineate cell-specific responses to exosomal cargo. Additionally, given the complexity of tumor-immune cell communication, further investigation is warranted to determine whether Texosomes modulate core intracellular signaling pathways, such as the PI3K/Akt/mTOR and Ras/MAPK axes that are essential for cell survival, proliferation, and effector function. Characterizing these signaling interdependencies will be a critical step toward understanding the molecular mechanisms by which tumor-derived vesicles promote immune exhaustion and tumor progression. Such insights will be vital for elucidating the precise mechanisms by which Texosomes reprogram the immune response during tumor progression and will provide a clearer understanding of their role as key regulators of anti-tumor immunity.

## 5 Conclusion

Collectively, our findings showed that exposure to 4T1-derived Texosomes is associated with altered splenocyte proliferation and NKG2D expression on NKp46^+^ splenocytes in vitro, supporting further investigation of Texosome-mediated immune modulation in tumor settings.

## CRediT authorship contribution statement

**Rizan Fallahzadeh:** Writing – original draft, Visualization, Software, Methodology, Investigation. **Motahare Khasteband:** Writing – original draft, Investigation. **Jafar Rezaie:** Supervision, Methodology. **Adel Mohammadzadeh:** Writing – review & editing, Validation, Supervision, Software, Conceptualization.

## Ethics declaration

This study was conducted in accordance with the ARRIVE (Animal Research: Reporting of In Vivo Experiments) guidelines. This study was approved by the Urmia University of Medical sciences. (Approval No. IR.UMSU.AEC.1400.147)

## Informed consent

Not applicable.

## Ethics statement

All animal care and experimental procedures were performed in accordance with the ethical guidelines approved by the Ethics Committee of Urmia University of Medical Sciences (Approval Code: IR.UMSU.AEC.1400.147). All authors acknowledge the ethical considerations relevant to this manuscript. This study adheres to the ARRIVE 2.0 guidelines to ensure the welfare of the experimental animals [36].

## Animal husbandry

Female BALB/c mice (6–8 weeks old, 21–23 g) were obtained from the Pasteur Institute of Iran (Tehran, Iran). The mice (n = 3) were acclimated under standard housing conditions with a controlled temperature (22 ± 2 °C) and a 12-h light/dark cycle. Animals had ad libitum access to food and water throughout the study. All procedures were conducted with appropriate environmental controls to minimize animal distress.

## Euthanasia and tissue collection

In accordance with Institutional Animal Care and Use Committee (IACUC) guidelines, mice were euthanized by carbon dioxide (CO_2_) inhalation at a flow rate of 3 L/min until respiration ceased. Death was confirmed by cervical dislocation. Following euthanasia, the carcasses were sterilized with 70% ethanol. The spleens were aseptically harvested using sterile instruments and immediately transferred into complete RPMI-1640 medium.

## Funding declaration

This research received no specific grant from any funding agency in the public, commercial, or not-for-profit sectors.

## Declaration of competing interest

The authors declare that they have no known competing financial interests or personal relationships that could have appeared to influence the work reported in this article.

## Data Availability

Data will be made available on request.
