# Supplementary material for: 4T1-derived exosomes downregulate NKG2D expression on NKp46+ splenocytes
Source: Biochem Biophys Rep. 2026 Nov 6;48:102874. doi: 10.1016/j.bbrep.2026.102874 (PMC13769550; doi:10.1016/j.bbrep.2026.102874)
Supplement: Supplementary file 1 [file mmc1.docx]

Supplementary Materials

All the necessary requirements all provided alongside the any picture to clear the readability. Please find the related information alongside the pictures. These are the analyzed data of the flow cytometery for the treated groups and untreated.

Here is the original pictures of the SEM and TEM for Texosomes:

Original picture of TEM for Figure 1 C (Down) , (S1B)

Original Picture of TEM for Figure 1 c (UP) (S1B)

SEM, Original Picture, Figure 1 A(S1A)

,

Original TEM picture result for Figure 1 B, (S1B)

Unstained Control

Single staining for NKG2D-PE , among the splenocytes

Single staining of The solenocytes for NKP46, Percp Cy5.5

Texosomes treated group gaited based on the lymphocytes and the results show the , NKP-Percp cy5.5 vs NKG2D-PE

Texosomes treated group gaited based on the lymphocytes and the results show the, NKP-Percp cy5.5 vs NKG2D-PE

Texosomes treated with Con-A group gaited based on the lymphocytes and the results show the , NKP-Percp cy5.5 vs NKG2D-PE

Texosomes treated with Con-A group gaited based on the lymphocytes and the results show the , NKP-Percp cy5.5 vs NKG2D-PE

Texosomes treated with Con-A group gaited based on the lymphocytes and the results show the , NKP-Percp cy5.5 vs NKG2D-PE

Texosomes treated with Con-A group gaited based on the lymphocytes and the results show the , NKP-Percp cy5.5 vs NKG2D-PE

Texosomes treated with Con-A group gaited based on the lymphocytes and the results show the , NKP-Percp cy5.5 vs NKG2D-PE

Texosomes treated with Con-A group gaited based on the lymphocytes and the results show the , NKP-Percp cy5.5 vs NKG2D-PE

Texosomes treated with Con-A group gaited based on the lymphocytes and the results show the , NKP-Percp cy5.5 vs NKG2D-PE

Con-A treated group gaited based on the lymphocytes and the results show the , NKP-Percp cy5.5 vs NKG2D-PE

Con-A treated group gaited based on the lymphocytes and the results show the , NKP-Percp cy5.5 vs NKG2D-PE

Con-A treated group gaited based on the lymphocytes and the results show the , NKP-Percp cy5.5 vs NKG2D-PE

Con-A treated group gaited based on the lymphocytes and the results show the , NKP-Percp cy5.5 vs NKG2D-PE

Con-A treated group gaited based on the lymphocytes and the results show the , NKP-Percp cy5.5 vs NKG2D-PE
